# Early Endothelial Activation in a Mouse Model of Graft vs Host Disease Following Chemotherapy

**DOI:** 10.3389/fimmu.2021.708554

**Published:** 2021-08-05

**Authors:** Melrine Pereira, Natasha Ting Lee, Jonathan Noonan, Abbey E. H. Willcox, Ilaria Calvello, Smitha Rose Georgy, Carly Selan, Joanne S. Chia, Wayne Hauw, Xiaowei Wang, Karlheinz Peter, Simon C. Robson, Harshal H. Nandurkar, Maithili Sashindranath

**Affiliations:** ^1^Australian Centre for Blood Diseases, Central Clinical School, Monash University, Alfred Hospital, Melbourne VIC, Australia; ^2^Atherothrombosis and Vascular Biology Laboratory, Baker Heart and Diabetes Institute, Melbourne, VIC, Australia; ^3^Department of Cardiometabolic Health, University of Melbourne, Melbourne, VIC, Australia; ^4^Department of Immunology, Monash University, Melbourne, VIC, Australia; ^5^Department of Anatomic Pathology, Melbourne Veterinary School, Faculty of Veterinary and Agricultural Sciences, The University of Melbourne, Werribee, VIC, Australia; ^6^Harvard Medical School, Department of Medicine, Division of Gastroenterology, Boston, MA, United States

**Keywords:** graft *vs* host disease, endothelial cell dysfunction, chemotherapy, mouse model, allogeneic haematopoietic stem cell transplant, allo-HSCT, monocrotaline, sinusoldal obstruction syndrome

## Abstract

Allogenic hematopoietic stem cell transplant (allo-HSCT) can lead to sinusoidal obstruction syndrome (SOS) and graft-versus-host disease (GvHD) in some individuals. GvHD is characterised by an immune triggered response that arises due to donor T cells recognizing the recipient tissue as “foreign”. SOS results in impaired liver function due to microvascular thrombosis and consequent obstruction of liver sinusoids. Endothelial damage occurs following chemotherapy and allo-HSCT and is strongly associated with GvHD onset as well as hepatic SOS. Animal models of GvHD are rarely clinically relevant, and endothelial dysfunction remains uncharacterised. Here we established and characterised a clinically relevant model of GvHD wherein Balb/C mice were subjected to myeloablative chemotherapy followed by transplantation of bone marrow (BM) cells± splenic T-cells from C57Bl6 mice, resulting in a mismatch of major histocompatibility complexes (MHC). Onset of disease indicated by weight loss and apoptosis in the liver and intestine was discovered at day 6 post-transplant in mice receiving BM+T-cells, with established GvHD detectable by histology of the liver within 3 weeks. Together with significant increases in pro-inflammatory cytokine gene expression in the liver and intestine, histopathological signs of GvHD and a significant increase in CD4+ and CD8+ effector and memory T-cells were seen. Endothelial activation including upregulation of vascular cell adhesion molecule (VCAM)- 1 and downregulation of endothelial nitric oxide synthase (eNOS) as well as thrombosis in the liver indicated concomitant hepatic SOS. Our findings confirm that endothelial activation is an early sign of acute GvHD and SOS in a clinically relevant mouse model of GvHD based on myeloablative chemotherapy. Preventing endothelial activation may be a viable therapeutic strategy to prevent GvHD.

## Introduction

Allogenic hematopoietic stem cell transplant (allo-HSCT) is used for the treatment of various haematological malignancies. It involves myeloablative conditioning (chemotherapy and radiation) to eliminate host immunity followed by the transplant of matched, and in some cases unmatched bone marrow stem cells to rescue the individual’s immune system. Despite its widespread use, patients undergoing allo-HSCT may develop several complications, including veno-occlusive disease or sinusoidal obstruction syndrome (VOD/SOS) and graft-versus-host disease (GvHD) as well as transplant-associated thrombotic microangiopathy, diffuse alveolar haemorrhage and capillary leak syndrome. Strikingly, all of these conditions are triggered by endothelial cell activation (1), and biomarker studies reveal that endothelial activation occurs across three phases after HSCT. The first phase is post-conditioning, the second occurs post-engraftment while the third occurs after onset of acute GvHD (aGvHD) (2).

GvHD is an adverse immunological response following allo-HSCT that develops in 30-60% of transplant recipients despite the use of standard prophylactics, making it a major cause of morbidity and mortality after allo-HSCT. It is an immune triggered response that results in exaggerated inflammation that arises due to donor T-cells recognizing the recipient tissue as “foreign”. aGvHD occurs in the first 100 days following allo-HSCT and mainly affects the skin, liver and gastrointestinal tract, manifesting as rash, diarrhea and weight loss (3).

As an active biological interface between the blood and tissues, cells constituting the vascular endothelium are affected first by the pre-HSCT conditioning regimen. Tissue injury caused by chemotherapy or conditioning induces release of pro-inflammatory cytokines including IL-1, IL-8, IL-2, and TNF-α, and IFN-γ and release of procoagulant molecules namely von Willebrand factor or vWF, thrombomodulin and plasminogen activator inhibitor-1. That endothelial activation occurs following chemotherapy in humans is supported by findings of increased presence of endothelial adhesion molecules cleaved and solubilised upon activation (e.g. E-selectin, intercellular cell adhesion molecule; ICAM-1 and vascular cell adhesion molecule; VCAM-1) (4). Conditioning results in both injury and activation of the vascular endothelium while post-transplantation, endothelial activation persists (5). Bacterial endotoxins that translocate through the intestinal mucosa after it is damaged due to conditioning, immunosuppressive therapy or engraftment promote pro-inflammatory cytokine release that aggravates endothelial toxicity (3). Further, because the vascular endothelium is the primary barrier separating the engrafting donor immune cells and GvHD target tissues, endothelial cells are effectively the first allogeneic cells encountered by primed T-effector cells (6). The activated endothelial cells upregulate MHCI expression, and as a result become targets for activated CD8+ cytotoxic T-cells during development of GvHD (7).

In humans, endothelial cell damage is seen after bone marrow transplant (BMT) and is exacerbated in those with aGvHD (2). Further, steroid refractory GvHD is associated with heightened endothelial dysfunction coinciding with T-cell activation (8). After allo-HSCT, patients who had aGvHD had higher bone marrow microvessel density together with increased VE-cadherin and vascular endothelial growth factor (VEGF) expression (9). A separate study showed that after allo-HSCT, vWF levels steadily increased from day 0 to day 21 while soluble VCAM-1 increased from day 7 to day 21 (10). Endothelial cells exposed to sera from aGVHD patients show increased VCAM-1 and ICAM-1 expression as well as a pro-thrombotic phenotype with vWF upregulation, all of which are reduced in the presence of defibrotide, the only drug currently available to treat SOS. This supports the hypothesis that aGvHD is due to systemic endothelial activation rather than an organ specific phenomenon (11).

In mice, endothelial cell apoptosis and vascular damage precede epithelial cell death in the oral mucosa in a model of aGVHD wherein immunodeficient mice are used and hence neither TBI nor chemotherapy-conditioning regimen is required (12). Endothelial damage is not limited to the microvasculature as it has also been shown to affect arterial vessels (13). Early GvHD in mice is associated with metabolic and cytoskeleton changes in endothelial cells of the gut and liver. These changes alter cell mechanics and promote endothelial proliferation causing angiogenesis, which coincides with GvHD onset (14). VCAM-1 and ICAM-1 are uniformly expressed in all GvHD target organs following allo-HSCT in mice, suggesting a strong role for these adhesion molecules in T-cell guidance in GvHD (15). Further, infusion of endothelial progenitor cells promotes hematopoietic and immune reconstitution, and reduces GVHD symptoms (6) while defibrotide inhibits endothelial cell proliferation and neo-angiogenesis that precedes the onset of aGVHD (16). Emerging literature continues to support a role for sustained endothelial damage in the initiation and promotion of aGVHD (17) and highlight that restoring endothelial function can be a potential therapeutic modality for GvHD.

Sinusoidal obstruction syndrome (SOS) of the liver results in swelling of endothelial cells, subendothelial oedema, red blood cell extravasation, fibrin deposition, and microthrombosis together with expression of factor VIII/vWF in venule walls. Liver function can be severely impacted; with the elevation of serum aminotransferase enzymes being a common occurrence after HSCT (18). SOS can occur independently of aGvHD, and this has been modelled in mice by Qiao et al. (19); but SOS also occurs in 14% of individuals undergoing allo-HSCT. During aGVHD, donor lymphocytes cause bile duct epithelial apoptosis, triggering endothelialitis and pericholangitis leading to dilation of the sinusoids and hepatocyte apoptosis and necrosis (20).

Mouse models of aGvHD generally utilise lethal total body irradiation (TBI) as conditioning typically without cytotoxic drugs. In contrast, most humans undergoing allo-HSCT are often subjected to chemotherapy conditioning with agents such as busulfan and cyclophosphamide which increases the risk of SOS. Because the type of tissue damage and pro-inflammatory responses that occur after conditioning can influence the nature of aGvHD, it is important to consider animal models in which myeloablative chemotherapy is used for conditioning (21). Accordingly, Riesner et al. (22) and Sadeghi et al. (23) both developed mouse models of aGVHD where busulfan/cyclophosphamide (Bu/Cy) were used for myeloablative conditioning. Following either minor (22) or major histocompatibility mismatched (23) stem cell transplants, clinically relevant aGvHD was detected in both models. Other than pronounced endothelium-mediated relaxation disrupting vascular barrier permeability in mice subjected to Bu/Cy treatment (24), to our knowledge, endothelial damage and dysfunction have not been studied in these models. Here we sought to characterise endothelial damage post-chemotherapy and allo-HSCT with the aim of providing rationale for future endothelial-targeted therapies to prevent onset and reduce target organ damage during GvHD.

## Methods

### Animal Model of Allo-HSCT

Experiments were approved by the A + (Alfred Research Alliance) Animal Ethics. Committee (Application E/1937/2019/M for GvHD and E/1748/2017/M for the toxin model of SOS).

Balb/C and C57Bl6 mice were from Monash Animal Research Platform (MARP) and were ~7-9 weeks old. Swiss Albino outbred mice Arc : Arc(S) were purchased from the Animal Resource Centre, Perth, Australia.

### Myeloablative Conditioning

Balb/C mice underwent myeloablative chemotherapy involving four days of Busulfan (days (d) -7 until d-4; 15mg/kg intra peritoneally (i.p.)) and two days of Cyclophosphamide (d-3 and d-2) 100mg/kg i.p), as published (22).

### Bone Marrow (BM) and Splenic T-Cells Preparation

BM was aspirated from the femurs and tibias and spleen harvested from adult C57Bl/6 mice. T-cells were isolated using the Pan T-cell Isolation Kit (Miltenyi Biotec, Australia) according to manufacturer’s instructions. Conditioned Balb/C mice were injected with 1 x10^7^ BM cells ± 4 x10^6^ splenic T-cells (intravenously (i.v.) *via* the tail vein) on day 0. Mice were euthanised on day 6 and 21 post-BM transplantation.

### Endothelial Toxin Model of SOS

SOS was induced in Arc : Arc(S) mice by i.p. injection of MCT (200 mg/kg) as described by Ikezoe et al. (25). This strain of mice is identical to the Japanese ICR mice used by Ikezoe et al. Animals were euthanised within 2 weeks of MCT injection *via* exsanguination and transcardial perfusion.

### Caspase-3 Assay to Determine Apoptotic Activity

Caspase activity was detected in liver and intestine lysates as described (26).

### Western Blotting

For detection of vWF, 2µl of plasma was electrophoresed and probed with sheep anti-vWF 1:1000; Abcam, MA, USA). Western blotting was carried out as published (26). For VCAM-1, a total of 40µg of protein from liver and gut lysates was resolved in 10% Mini-PROTEAN TGX Stain-Free Protein Gels (Bio-Rad Laboratories Pty Ltd, Australia) and rabbit anti-VCAM-1 (1:1000; Abcam, USA) and rabbit anti-eNOS (1:500; Abcam, USA) was used. For plasma von Willebrand Factor (vWF) analyses, 2µl of sample were resolved in 7.5% polyacrylamide gels, and sheep anti-vWF 1:1000; Abcam, MA, USA) was used to detect vWF. A standard curve was created using known concentrations of recombinant vWF assessed *via* western blot, and the amount of vWF was calculated from that curve.

### Immunofluorescence Identification of Endothelial VCAM-1

Frozen liver sections (10µm) were incubated with rabbit anti-VCAM-1 (1:200) or rabbit anti-cleaved caspase-3 Asp175 (5A1E) (1:100, Cell Signalling Technology USA) and sheep anti-von Willebrand Factor (vWF; 1:100) (Abcam, MA, USA) overnight at 4°C. Alexa Fluor^®^ 647 Donkey anti-Rabbit IgG (H+L) and Alexa Fluor 488 Donkey anti-Sheep IgG (H+L) (1:900; Thermo Fisher Australia) were used as secondary antibodies. Images were captured using the Nikon TiE microscope (Monash Micro Imaging Platform).

### Histology

#### Haematoxylin and Eosin and Carstairs Staining

Formalin fixed paraffin embedded intestine, liver and skin (from ear) sections (aGvHD and MCT models; 4µm) were stained with H&E as described (27). To assess histopathological signs of GvHD, liver sections were scored for portal inflammation, sinusoidal lymphocytosis, vascular endothelialitis, parenchymal mitotic figures, bile duct lesions and bile duct epithelial cell sloughing as published by Cooke et al. (17). Histopathological changes in the liver, intestine and skin were quantified in randomly selected 20x micrographs from 3 different 5 µm sections (10 images per mouse) by a 0-3-point score by a trained pathologist blinded to experimental groups. Liver sections stained with Carstairs stain by the Monash Histology Platform; Carstairs staining was quantified as published (28).

### Liver Function Tests

Liver function enzymes in citrated plasma were assessed using a clinical biochemical analyser at the Monash Pathology service.

### Real-Time PCR

RNA was isolated from liver and intestine tissue samples using ReliaPrep™ RNA Tissue Miniprep columns (Promega, Australia) and cDNA prepared using the SensiFAST™ cDNA Synthesis Kit (Bioline (Aust) Pty Ltd, Australia) according to manufacturer protocols. Changes in expression of GAPDH (Mm99999915_g1), IL-1α (Mm00439620_m1), IL-1β (Mm00434228_m1), IL-2 (Mm00434256_m1), IL-6 (Mm00446190_m1), IL-18 (Mm00434226_m1), TNFα (Mm00443258_m1) IFNγ, VCAM (Mm01320970_m1), were assessed using TaqMan^®^ Gene Expression Assays (Thermo Fisher Scientific, Australia) and the SensiFAST Probe No-Rox Kit (Bioline (Aust) Pty Ltd, Australia) as described (26).

### Flow Cytometry

Flow cytometry was performed on liver samples as described (29) using antibodies listed in **Table 1**. Samples were analysed using the BD LSRFortessa™ X-20 Cell Analyzer (BD Biosciences Australia).

**Table 1:** Antibody cocktail T cell panel for flow cytometry.

[B] 530/30	BB515 CD62L 1:300
[R] 780/60	APC-Cy7 CD45 1:200
[YG] 780/60	PE-Cy7 H-2Kd 1:200
[UV] 379/28	BUV395 CD8 1:300
[UV] 525/50	BUV496 CD4 1:400
UV] 740/35	BUV737 CD44 1:200
[V] 525/50	BV510 CD3 1:100
[V] 780/60	BV786 H-2Kb 1:200

### Statistical Analysis

Statistics analyses were performed using GraphPad Prism version 8.0 (GraphPad Software Inc). Tests are described in the figure legends.

## Results

**Figure 1:**
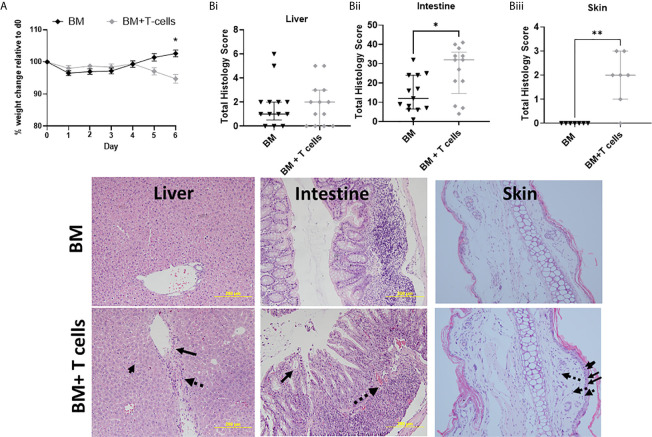
Onset of GvHD at day 6 post-transplantation after myeloablative chemotherapy- Significant differences in weight and high histological scores indicate the onset of GvHD at day 6 in mice transplanted with BM+T-cells compared to mice transplanted with BM only. **(A)** Post-transplant, significant difference in weight are observed at day 6 between mice transplanted with BM and BM+T-cells (*p<0.05). Two-Way RM ANOVA with Sidak’s multiple comparison test. Data is Mean ± SEM. n=13. **(B)** Mice transplanted with BM+T-cells showed **(i)** no change in GVHD Histology score compared with animals transplanted with BM only in the liver and (image) although representative sections show apoptotic bodies (small solid arrow), endothelialitis (large solid arrow) and lymphocytic infiltrate (dotted arrow). However **(ii)** increased GvHD score was seen in the intestine samples from the BM+ T cells cohort as evidenced by (image) representative histology images showing crypt destruction (solid arrow) and inflammatory infiltrate (dotted arrow). **(iii)** Cutaneous GvHD is also evident in this model with abundant apoptotic/necrotic keratinocytes (solid arrows) and intradermal lymphocyte infiltration (dotted arrows) *p < 0.05. Unpaired Mann-Whitney U-test. Data is median with interquartile range and n=3-6 naïve; n=12-13 BM and BM+T cells (liver and intestine) and n=7 (skin). **p < 0.01.

Weight loss is a clinical sign of GvHD. During the first 9 days (from day -7 to day 1) all mice, irrespective of their treatment group showed weight loss (**Figure 1A**). However, post-transplant beginning at day 4 until day 6, weight loss was sustained in mice that underwent BM+ T-cell transplant, with significant difference in weight observed at day 6 (**Figure 1A**). This data indicates the onset of GvHD at day 5-6 post-transplant.

Although there was a trend for higher mean GvHD histological scores at day 6 post BM+T-, these results were not significant at this stage (**Figure 1Bi**). Lymphocytosis, endothelialitis and portal inflammation were observed in the BM+T cells cohort (**Figure 1** Image). In contrast, significantly higher mean histopathological scores were observed in intestines of mice at day 6 post BM+T-cells compared to mice transplanted with BM only (**Figure 1Bii**). Prominent crypt destruction and inflammation was observed (**Figure 1** Image). Increased keratinocyte cell death in the epidermis and pronounced dermal lymphocytic infiltrate confirmed histopathological GvHD in the skin at this timepoint (**Figure 1Biii**).

**Figure 2:**
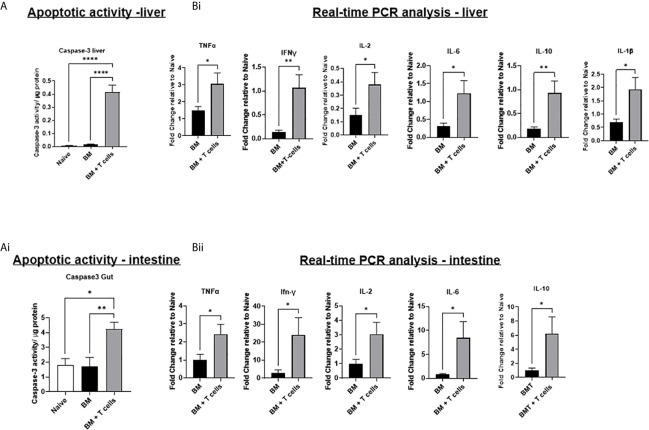
Significant increases in apoptotic activity and in gene expression of inflammatory cytokines suggests the onset and progression GvHD in mice transplanted with BM+T-cells –**(A)** Significant increase in caspase-3 activity in the **(i)** liver and **(ii)** intestine of mice transplanted with BM+T-cells compared to mice transplanted with BM only. A fluorescence-based caspase 3 activity assay was used to determine apoptotic activity in liver and intestine lysates. Data is Mean ± SEM. n=5-7. *p < 0.05, **p < 0.005, ****p < 0.00005. One-way ANOVA with Tukey multiple comparison Test. **(B)** Quantitative real time PCR analysis showing significant increases in gene expression of inflammatory cytokines in **(i)** liver and **(ii) **intestine samples of mice transplanted with BM+T-cells compared to BM only. Data is Mean± SEM. n=5-7. *p < 0.05. Unpaired t test.

Despite no evidence for a significant increase in histopathological GvHD in the liver at this timepoint, apoptotic activity was significantly increased in both the liver and intestine of mice transplanted with BM+ T-cells (**Figure 2A**). No significant increase in apoptotic activity was observed in the liver and gut of mice transplanted with BM only. These results suggest that apoptosis in the liver and intestine are indicative of GvHD onset rather than chemotherapy induced toxicity. Increased gene expression of all pro-inflammatory cytokines (TNF-α, IFN-γ, IL2, IL6 and IL10) in the liver and intestine, as well as IL-1β in the liver of mice transplanted with BM+T-cells further confirms this (**Figure 2B**). These results suggest the development and progression of GvHD in mice transplanted with BM+T-cells.

**Figure 3:**
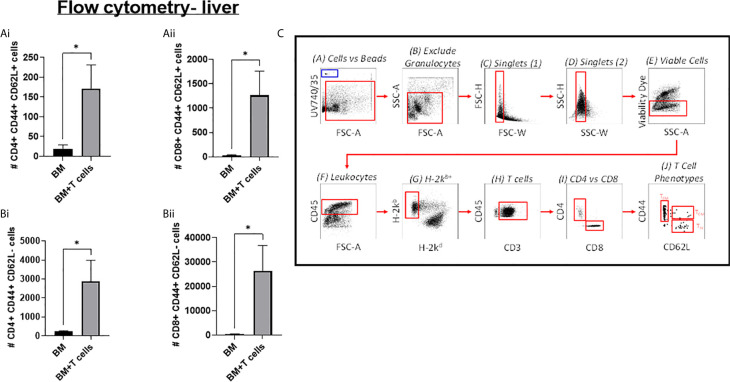
Significant increases in T-cells in the liver at day 6 post-GvHD- Flow cytometry analysis shows significant increases in effector and central memory T-cells **(A) (i)** CD4+ and **(ii)** CD8+ TEM (CD44+ CD62L+) and **(B) (i)** CD4+ and **(ii)** CD8+ TCM (CD44+ CD62L-)cells in the liver confirms the onset and progression GvHD in mice transplanted with BM+T-cells. **(C)** Gating strategy for flow cytometry analysis. Data is Mean ± SEM. n=9. *=p < 0.05 unpaired Students t-test.

Indeed, GvHD onset was supported by flow cytometry data showing that mice transplanted with BM+T-cells had a significantly higher population of CD4+ T-effector memory cells (T_EM_) (H2Kb+ CD4+ CD44+CD62L-) and CD8+ T_EM_ (H2Kb+ CD8+ CD44+CD62L-) in the liver (**Figure 3A**). We also found that mice transplanted with BM+T-cells had a significantly higher population of central memory T- cells (T_CM_), both CD4+ T_CM_ (TcM) (H2Kb+ CD4+ CD44+CD62L+) and CD8+ T_CM_ (H2Kb+ CD8+ CD44+CD62L+) in the liver (**Figures 3B, C**).

**Figure 4:**
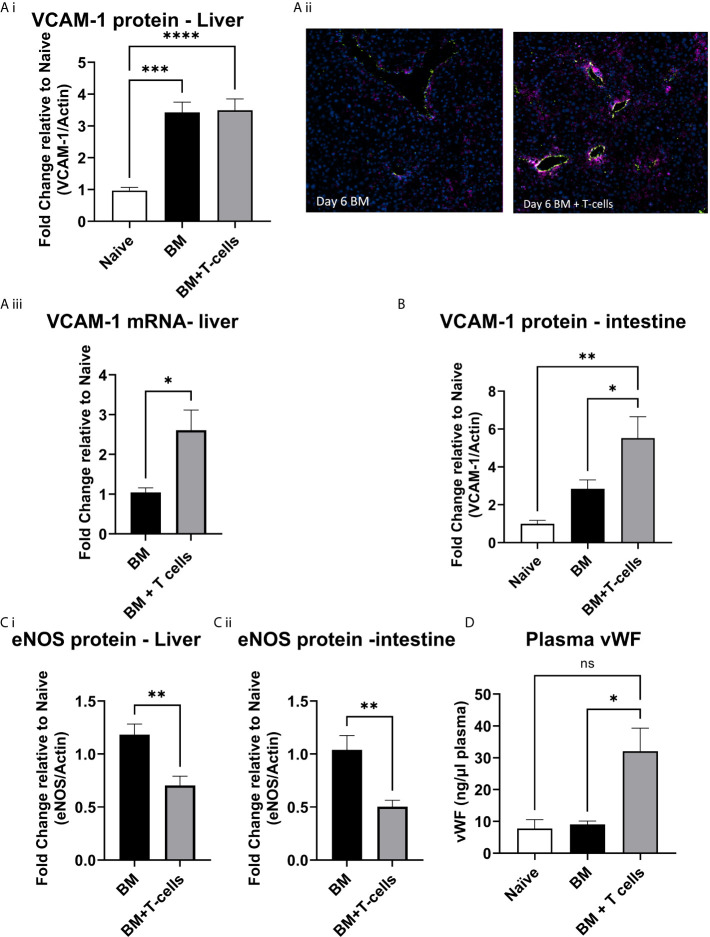
Endothelial activation at day 6 post-GvHD- **(A) (i)** VCAM protein levels are increased in the liver at day 6 post BM and BM+T-cell transplant. ***p < 0.0001, n=7 naïve and n=13 BM and BM +T cells but **(ii)** endothelial VCAM-1 is increased selectively in the BM+T-cell transplant cohort (Immunostaining with anti-Von Willebrand Factor (green) to delineate the endothelium, anti-VCAM (purple) and DAPI to identify nuclei. Representative images of N=2 sections per mouse and n=3-4 mice per group. **(iii)** VCAM mRNA expression is higher in the BM+T cell cohort and **(B)** VCAM protein levels are significantly higher in the intestine of mice undergoing BM+T cells transplant. One Way ANOVA, with Sidak’s multiple comparison test. **(C)** Endothelial nitric oxide synthase (eNOS) is reduced in **(i)** liver and **(ii)** intestine confirming increased endothelial damage among mice transplanted with BM+T-cells. **p < 0.005; unpaired t test, n=6. **(D)** Plasma vWF is significantly elevated in the BM+T-cells cohort at day 6 post-transplant. *p < 0.05; one-Way-ANOVA with Tukey’s multiple comparison test. n=3 naïve; n=13 BM and BM+T cells. Data is densitometric quantification relative to actin; Mean ± SEM. ****p < 0.001; NS, not significant.

A significant increase in VCAM-1 expression was observed in both groups of mice (BM and BM+T-cells) suggesting an effect of chemotherapy rather than GvHD (**Figure 4Ai**). However, mRNA expression of VCAM-1 was significantly higher in the latter group (**Figure 4Aii**) indicating that GvHD related VCAM-1 upregulation had been triggered. This increase is perhaps not seen as yet in the VCAM protein levels as transcription would naturally precede translation. Nevertheless, endothelial expression of VCAM-1, studied with vWF/VCAM-1 double immunofluorescence in the liver was seen only in the BM+T cells cohort (**Figure 4B**). Therefore, endothelial activation coincides with T-cell activation and apoptosis in the liver at this timepoint. In the intestine samples, significant increases in VCAM-1 were observed in mice transplanted with BM+T-cell compared to mice transplanted with BM only and control naïve mice (**Figure 4C**).

Mice transplanted with BM+T-cells showed decreased eNOS expression in both the liver and gut compared to mice transplanted with BM (**Figures 4Ci, ii**). Furthermore, an overall increase in plasma levels of vWF (**Figure 4D**) strongly corroborates our hypothesis that early endothelial damage occurs simultaneously with GvHD onset in our model.

**Figure 5:**
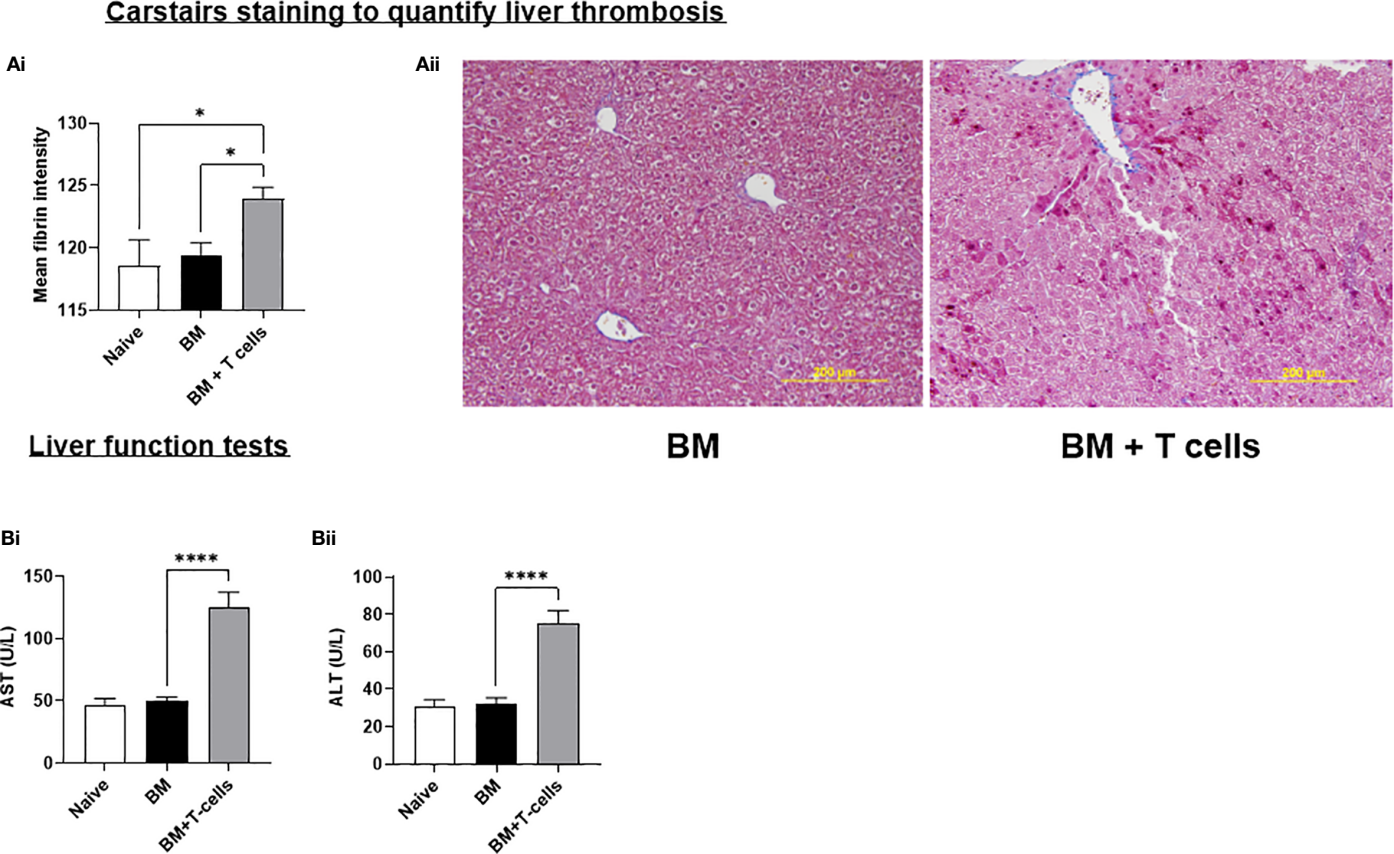
Hepatic SOS is evident at day 6 post-GvHD- **(A) (i)** Graph depicts mean intensity of Fibrin using Fiji/Image J * **(ii)** Representative Carstairs stain images show liver sections, sectioned at 4µm where blue depicts collagen, dark red- Fibrin and yellow-red blood cells. **(B)** Liver Function Test shows increased levels of **(i)** aspartate aminotransferase (AST) and **(ii)** alanine aminotransferase (ALT) which is commonly associated with SOS. *p < 0.05, ****p < 0.00005, n=6 naïve; n=13 BM and BM+T cells. Data is densitometric quantification relative to actin; Mean ± SEM. One-Way ANOVA, Tukeys *post hoc* analysis.

We next investigated whether this endothelial damage would cause SOS in the liver. Carstairs stained sections were digitally quantified to reveal significant increases in mean fibrin staining in mice transplanted with BM+T-cells (**Figure 5A**). These results are consistent with signs of SOS developing in this model and also show that microvascular thrombosis precedes the histopathological GvHD in the liver in this model. SOS in our model was further confirmed by elevated alanine aminotransferase (ALT) and aspartate aminotransferase (AST) in mice transplanted with BM+T-cells (**Figure 5B**). Taken together, we have strong evidence to suggest that our model accurately recapitulates SOS seen in patients with GvHD and that endothelial dysfunction is a strong predictor of both GvHD onset and SOS in this pre-clinical model.

**Figure 6:**
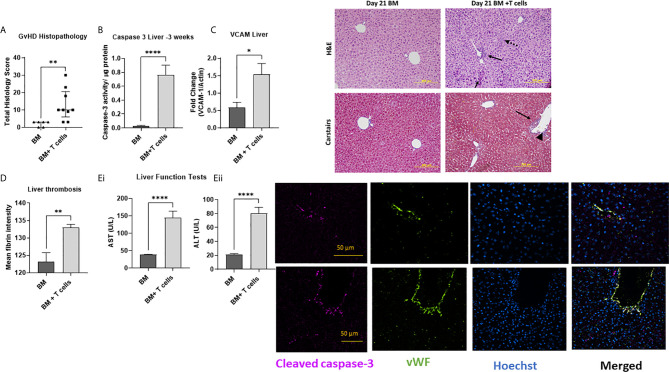
Established GvHD, endothelial and hepatic SOS at d21 post-transplant of BM + T-cells **(A)** with increased histopathological signs of GvHD (Data is median and interquartile range, *=p<0.05 Mann-Whitney U-test. **(B)** Augmented caspase-3 activity confirms significant liver damage and **(C)** VCAM-1 upregulation. Hepatic SOS is evidenced by **(D)** increased fibrin deposition and **(E)** impaired liver function with increased levels of **(i)** aspartate aminotransferase (AST) and **(ii)** alanine aminotransferase (ALT) are also noted. **p < 0.005, ****p < 0.00005, unpaired Student’s t-test, n=6 naïve; n=6 BM and n=9 BM+T cells. Data is Mean ± SEM. Image: H&E images show periportal inflammation (solid arrows) and sinusoidal infiltration (dotted arrows) which Carstairs image (blue depicts collagen, dark red- Fibrin and yellow-red blood cells) shows abundant fibrin deposition with parenchymal mitotic figures (long arrow) and vascular endothelialitis (arrowhead) remaining visible. Immunofluorescence staining shows specific cleaved caspase-3 immunoreactivity colocalizing with endothelial cells positive for vWF in both large and small vessels, with Hoechst used as a nuclear counter-stain. Merged images confirm presence of apoptotic endothelial cells.

To study how these changes at d6 impacted GvHD onset, we studied a cohort of mice at d21 post- BM ± T-cells. We found that unlike at d6, histopathological GvHD was evident at d21 (**Figure 6A**) with significant increases in periportal inflammation and lymphocyte infiltration with increased caspase-3 activity (**Figure 6B**) and VCAM-1 upregulation (**Figure 6C**) affirming that increased mRNA levels of VCAM-1 preceded increased translation of the molecule. Increased fibrin deposition (**Figure 6D**) and significantly elevated aminotransferase enzymes further confirmed impaired hepatic function indicating clinical signs of SOS (**Figure 6E**). Immunofluorescence-based colocalisation of endothelial vWF with cleaved caspase-3 revealed a strong presence of apoptotic endothelial cells, further validating that protracted endothelial damage is seen in this model (**Figure 6** Image).

**Figure 7:**
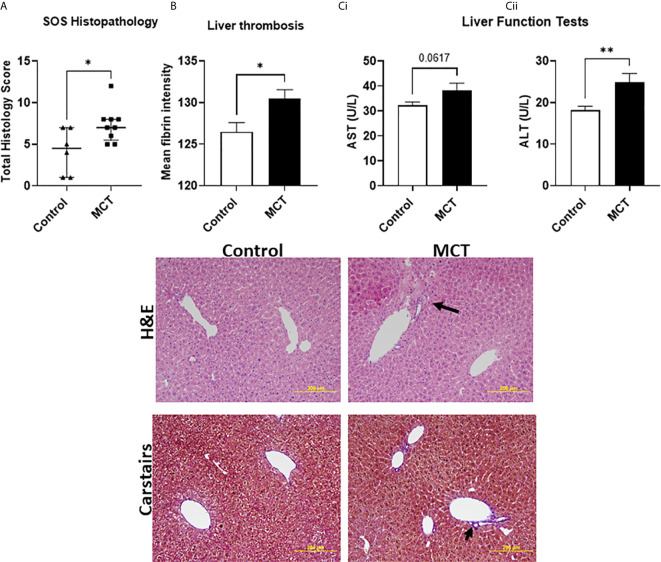
SOS can be directly induced with monocrotaline-mediated endothelial toxicity: **(A)** Increased hepatic damage reported *via* histological analysis (Data is median and interquartile range, *=p < 0.05 Mann-Whitney U-test) and **(B)** increased thrombosis in the liver as well as **(C) (i)** a trend towards increased aspartate aminotransferase (AST) and **(ii)** significantly increased alanine aminotransferase (ALT) levels confirming pronounced hepatic damage.). *p < 0.05, **p < 0.005, unpaired Student’s t-test; n=7 BM and BM+T cells. Data is Mean ± SEM.

As a proof of concept that endothelial dysfunction is an underlying cause for SOS in our model of aGvHD, we used the endothelial toxin model monocrotaline to chemically induce SOS. In this model, SOS occurs independent of T-cell activation (25). Histopathological assessment revealed significant necrotic zones, hepatocellular mitotic figures and vascular endothelialitis (**Figure 7A** and image). Quantification of fibrin rich areas detected by Carstairs staining revealed significantly increased fibrin deposition in mice receiving MCT (**Figure 7B** and image). Following MCT injection, mice had increased ALT (p=0.06) and significantly increased AST levels (**Figures 7Ci, ii**), similar to mice that had aGvHD.

## Discussion

The allo-HSCT model we have used is a clinically relevant model of GvHD that offers valuable insights into the cumulative effects of chemotherapy and allo-HSCT in target organs. Here we studied the early effects of allo-HSCT on GvHD target organs.

In a minor mismatch -chemotherapy based GvHD model wherein 2x 10^6^ splenic T-cells were transplanted, significant weight loss and therefore GvHD onset was detected from day 15 onward. Histopathological signs of GVHD were also evident at this timepoint (22). Although a strong indicator of GvHD in mice, weight loss also occurs after chemotherapy with Bu/Cy (23). Similar to our findings, in this published model, mice receiving allo-HSCT displayed the highest degree of weight loss at day 7. Although we have not compared the TBI and chemotherapy methods of myeloablation in this study, the paper by van Leeuwen et al. (30) shows that in terms of GvHD onset the two models show similar temporal profiles. After TBI and transfer of 5x10^6^ splenic T cells from C57Bl6 mice to Balb/C mice, these mice had weight loss over two phases, with the first phase reaching its nadir at day 7 (30). We also saw increased apoptotic activity in both the liver and intestine at day 6 and in the liver at d21 with prominent apoptotic endothelial cells noted. Blinded histological scoring revealed increases in both crypt apoptosis in the gut. This further confirms GvHD onset at this timepoint. That histopathological signs of GvHD were significantly increased in the intestine and not in the liver at d6 may indicate that the combination of chemotherapy and T-cell transplant has a more acute response in the intestine in this model while in the liver distinctive GvHD-related cell damage evolves 3 weeks post-transplant.

The BM+ T cells group had increased mRNA expression of pro-inflammatory cytokines TNFα, IFN- γ, IL-2, IL-6 and IL-10 in both liver and intestine samples and expression of IL-1β was also increased in the liver at day 6. Of these, TNFα, IFN- γ, IL-1β and IL-6 are known to be triggered during the cytokine storm and release of microbiota derived DAMPs and PAMPs following chemotherapy. IFN-γ, IL-2, and TNFα are generated during differentiation into a T helper 1 response during aGvHD (31). Sadeghi et al. used a similar model involving HSCT preceded by Bu/Cy conditioning and showed increased serum levels of IFN-γ, TNFα and IL-2 at day 5, with concomitant clinical signs of GvHD (32). Similar serum levels of IFN-γ, TNFα were also reported at day 8 post- chemotherapy with minor mismatched transplant (22).

Endothelial activation is primarily mediated *via* pro-inflammatory cytokines TNFα and IL-1β bind to their respective receptors on endothelial cells, thereby triggering activation NFκb. This and increased expression of adhesion molecules including VCAM-1 (33). Increased expression of VCAM-1, E-, and P-selectin is associated with established GVHD while significant downregulation of gene expression levels of these markers is seen at the early stages post-transplant in mice (14, 15). Our results showing significant increases in VCAM-1 in the gut are akin to these data.

Endothelial dysfunction in GvHD target organs in our model was further confirmed by reduced eNOS levels. Reports show eNOS expression is decreased in mesenteric arteries in a similar C57Bl6➔ Balb/C mouse model albeit based on myeloablative conditioning with TBI. This further indicates that endothelial dysfunction post-allo HSCT occurs after both TBI and chemotherapy based myeloablative conditioning. Decreased eNOS expression strongly correlated with ultrastructural damage to the endothelium of the mesenteric arteries, characterised by gaps in between endothelial cells, cell detachment and death (13).

vWF is a reliable marker of endothelial damage, particularly in the context of hepatic SOS (34). Buser et al. showed that allo-HSCT patients have higher plasma vWF levels, and those with GVHD had significantly higher vWF levels than those without (35). Biedermann et al. reported that cytotoxic (CD8+) T cell infiltrates in skin samples of GvHD patients correlated with vWF plasma concentrations (36). Accordingly, we found a significant increase in donor CD4 and CD8 effector memory T-cells in the liver, consistent with Riesner et al. who showed that similar increases in peripheral blood and lymph nodes at day 15. Whether concomitant T-cell infiltration also occurs in the intestine remains to be confirmed, although CD3+ cells in the intestine were detected with immunofluorescence (not shown). Collectively, these data confirm that host endothelial cells are activated and release vWF during GvHD onset as they are a target of the infiltrating alloreactive donor T lymphocytes.

SOS causes decreased liver blood flow leading to prolonged hypoxemia and decreased oxygenation of hepatocytes which can be diagnosed by a rapid rise in serum AST and ALT (18). Elevated aminotransferases also defines a subset of hepatic GvHD (37). A ~2-3x elevation in both AST and ALT at day 6 in mice that received the BM+T cells infusion points to clinical signs of hepatic GvHD in this group. Unlike the paper by Qiao et al, we did not see altered enzyme levels in mice receiving chemotherapy alone (19). Carstairs staining is routinely for histological detection of platelets/fibrin/collagen/red cells in thrombi (28). Automated image quantitation of the fibrin-rich areas revealed a significant increase in fibrin deposits in the BM+T cells cohort. Although we have not quantified perivenular oedema, sinusoidal dilatation, extravasated red cells or collagen/platelet staining, these were also evident (**Figure 5Aii**). Therefore, early hepatic GvHD and SOS coincide with endothelial activation in our model. MCT causes injury to the central venous and sinusoidal endothelial cells, leading to centrilobular hepatocellular necrosis, sinusoidal congestion and dilatation, and haemorrhage. In showing that endothelial damage causes SOS and the similarities in the histopathology, augmented liver enzyme levels and thrombosis of the liver, we were able to validate that our aGvHD model accurately recapitulates SOS seen in a subset of GvHD cases.

There is strong criticism of translational studies in the field of HSCT, as chemotherapy-alone regimens are rarely studied in mouse models of aGVHD (38). While our model addresses this criticism, the caveat of using major MHC-mismatch strains has to be acknowledged, as these strains often differ in their T-cell responses (38). Although we have cited references showing that it is likely to be similar, it would be useful to compare and contrast the evolution of endothelial dysfunction after allo-HSCT following both TBI and chemotherapy. Nonetheless, that our model accurately recapitulates many aGvHD features seen in humans is evidenced by findings pertaining to endothelial dysfunction. In humans, endothelial permeability and inflammatory and thrombotic responses of the endothelium causing cell death are important initial events occurring within the first 2 months after allo-HSCT, including GvHD. This issue is further compounded by the direct and indirect effects of conditioning and the effect of the GvHD-induced immune response on the endothelium (34). Having focussed entirely on the early effects on the endothelium, we will now focus on the evolution of aGvHD in our model by studying the model over 28-35 days.

Endothelial damage is the first hit in the process that is then triggered by immunological response from donor T cells. Although T-cell depletion of allografts would be the logical path to avoid GvHD, most of these approaches have resulted in a higher risk of infection, reduced donor T-cell–mediated graft-versus-leukemia effect, delayed immune reconstitution and a higher risk of relapse (39). Targeting endothelial damage early after allo-HSCT has therapeutic potential to prevent or alleviate GvHD. Indeed, Garcia-Bernal et al. recently described the effects of defibrotide following aGvHD induction with TBI and C57Bl6➔ BalbC allogeneic transplant. Defibrotide-treated recipient mice had improved clinical scores as well as reduced T-cell infiltrates in the skin and liver. Further, prophylactic daily administration of defibrotide minimised inflammatory responses, supporting our hypothesis that treating endothelial dysfunction can reduce aGvHD (16). Moreover, there are no laboratory tests available to predict risk of developing GvHD after allo-HSCT. Improved understanding about the link between endothelial dysfunction and GvHD might help identify markers of endothelial damage as predictors of GvHD, thereby filling this gap in access to clinical biomarkers for GvHD (7).

## Data Availability Statement

The original contributions presented in the study are included in the article/supplementary material. Further inquiries can be directed to the corresponding author.

## Ethics Statement

The animal study was reviewed and approved by A + (Alfred Research Alliance) Animal Ethics Committee (Application E/1937/2019/M and E/1748/2017/M).

## Author Contributions

MP, NL, JN, AW, IC, CS, JJ, and SG - experiments, data collection, analysis, and manuscript editing. XW, KP, and SR -research direction and critical evaluation of manuscript. MS - experiments, data collection, analysis, and manuscript writing. HN - experiment planning, critical evaluation of manuscript, manuscript editing and project funding. All authors contributed to the article and approved the submitted version.

## Funding

This work was supported by NHMRC Project grant APP1141046 awarded to HN.

## Conflict of Interest

The authors declare that the research was conducted in the absence of any commercial or financial relationships that could be construed as a potential conflict of interest.

## Publisher’s Note

All claims expressed in this article are solely those of the authors and do not necessarily represent those of their affiliated organizations, or those of the publisher, the editors and the reviewers. Any product that may be evaluated in this article, or claim that may be made by its manufacturer, is not guaranteed or endorsed by the publisher.
